# *Operando* XPS studies of precisely size-selected Pd nano-catalysts for methane oxidation

**DOI:** 10.1039/d5fd00171d

**Published:** 2026-03-18

**Authors:** Alexander I. Large, Henry Hoddinott, Haamidah Sana, Elizabeth Jones, James J. C. Counter, Matthijs A. van Spronsen, Santosh Kumar, David C. Grinter, Pilar Ferrer, Bernd von Issendorf, Richard E. Palmer, Georg Held

**Affiliations:** a Diamond Light Source, Harwell Science Campus Didcot OX11 0DE UK georg.held@diamond.ac.uk; b Swansea University UK; c Oxford University UK; d University of Reading UK; e Albert-Ludwigs-Universität Freiburg im Breisgau Germany

## Abstract

The importance of cluster-size effects in heterogeneous catalysis is now well recognized. X-ray photoelectron spectroscopy (XPS) is an obvious technique to study size-dependent changes in the chemical composition and electronic structure of catalyst nanoparticles. However, as XPS is an averaging technique based on the detection of electrons, experiments require a narrow distribution of cluster size and a conducting homogeneous support in order to avoid sample charging, which would prevent accurate measurements of chemical shifts. Traditional methods of catalyst synthesis by impregnation/calcination of support powders lead to very large particle size distributions (typically ±50%) and insulating samples. They therefore fail both of the above criteria and make it extremely difficult to extract precise sample characterisation. Here we present an alternative approach designed to enable XPS analysis in vacuum and under reaction conditions, whereby: (i) nanoparticles are synthesized by gas condensation and passed through a mass filter, which allows size selection in the range of 1 to 10 000 atoms with typically ±4% accuracy; (ii) these particles are deposited onto a thin Al_2_O_3_ film grown on Al foil, which mimics the properties of conventional alumina supports while being conductive enough to avoid any charging-related artefacts in the XPS spectra. In vacuum, size-dependent Pd 3d binding-energy shifts up to 1.65 eV were recorded for supported Pd nanoparticles. Changes in the chemical composition of Pd nanoparticles were studied by near-ambient pressure (NAP)-XPS under dry and wet reaction conditions for methane oxidation (CH_4_ + O_2_ [+H_2_O]) in the temperature range between 150 °C and 450 °C. Under dry reaction conditions large Pd particles appeared to oxidise almost fully to Pd(ii), whereas smaller clusters showed a mix of Pd(0) and Pd(ii) oxidation states. Under wet conditions, oxidation starts at lower temperatures and particles of all sizes were fully oxidised when the highest temperature was reached. Sintering during the temperature ramp cannot be excluded, especially for the smaller particles, and may be part of the reason for the different behaviour under wet conditions. This study clearly shows composition changes which are particle-size dependent and demonstrates the possibilities of fine-tuning catalytic activity if better size-control can be achieved in catalyst synthesis.

## Abstract 2

Changes in the chemical composition of size-selected Pd nanoparticles were studied by near-ambient pressure XPS under dry and wet reaction conditions for methane oxidation (CH_4_ + O_2_ [+H_2_O]) in the temperature range between 150 °C and 450 °C.

## Graphical abstract

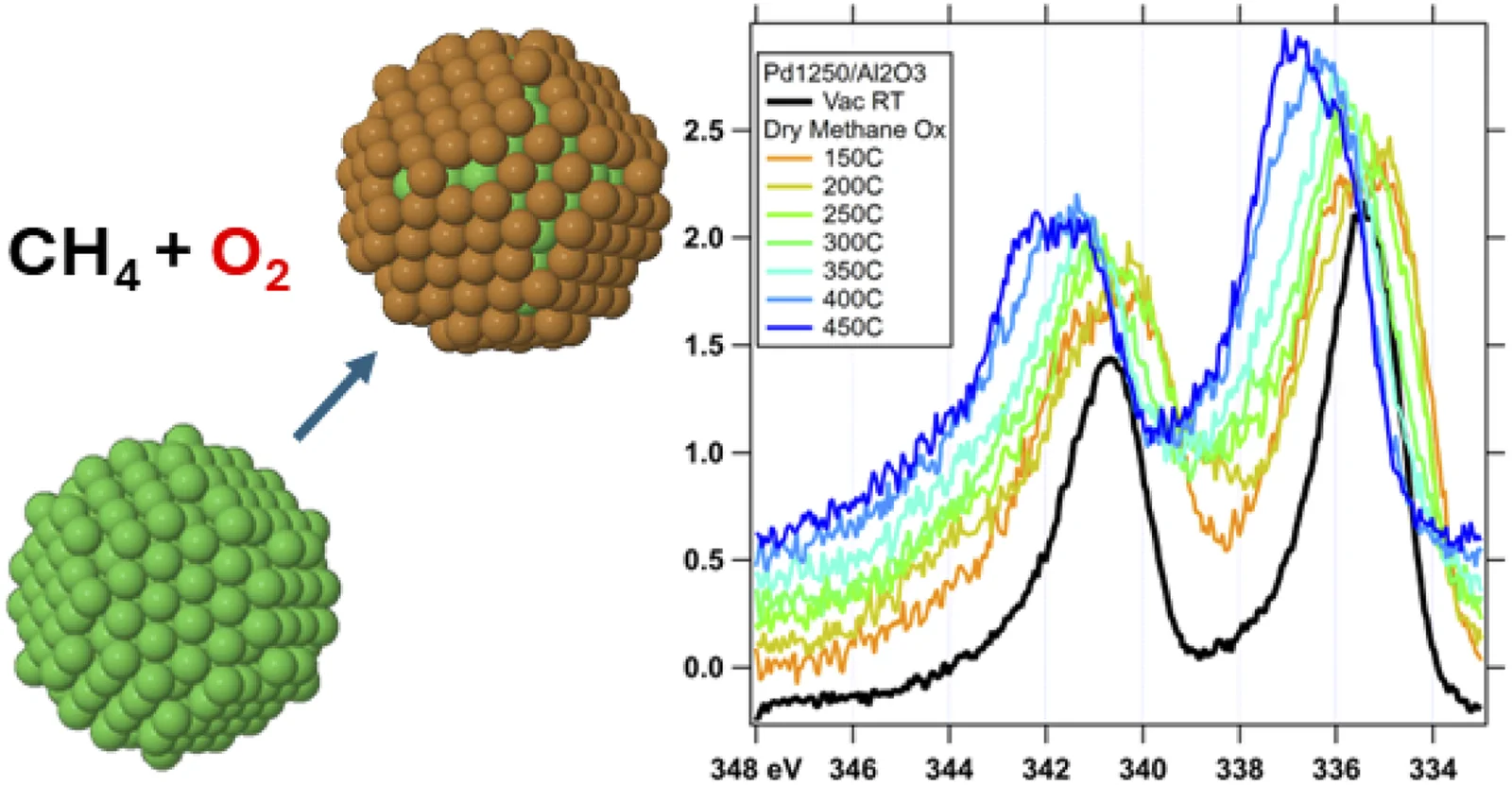

## Introduction

The importance of both particle size and catalyst-support interaction in heterogeneous catalysts has been recognised for some time.^1–5^ In particular, in the size range between <1 nm and 5 nm, many catalyst materials show dramatic changes, sometimes at the level of orders of magnitude, in activity and selectivity.^6–9^ One way of varying the particle size is to deposit size-selected clusters on surfaces and thus create model catalysts. Catalytic studies of such systems began in the 1990s, typically under ultra-high vacuum with gas pressures below 10^−6^ mbar. Reactions included Fischer–Tropsch and CO oxidation.^10,11^

The volume of work on size-selected clusters is very small compared with a much larger body of research studying particle size and support effects in catalysts synthesised under industrial conditions, with relatively poor size control. Typically measured are macroscopic parameters, such as activity and selectivity of supported catalyst powders. As things stand, there is a significant gap between these two kinds of catalytic research, *i.e.* size-selected clusters and catalyst powders. Only a small body of published work investigates the size-dependent chemical behaviour of nanoparticles under realistic reaction conditions, *e.g.* ref. 12–15. In this regime surface-sensitive spectroscopic methods have much to offer. Here we report on such a study, using near-ambient pressure X-ray photoelectron spectroscopy (NAP-XPS) coupled with size-selected cluster deposition on thin conducting oxide supports. Specifically, we determine the chemical state of alumina-supported Pd nanocatalysts during the methane oxidation reaction.

In standard powder catalyst preparation, the main parameter used to control particle size is the calcination temperature post-impregnation, but the result tends to be a broad distribution of sizes, often more than 50% of the mean size, with outliers ranging from single atoms to >10 nm,^16–20^ which can seriously affect the validity of conclusions. For clear understanding of size effects, it is therefore essential to prepare nanoparticles with narrow size distributions, which requires much greater control than achievable with calcination. One way of improving the size distribution of nanoparticles is through colloidal synthesis,^3^ however this method necessitates the removal of the colloid ligands after the synthesis if pure metal catalysts are required. Cluster beam deposition is an alternative, solvent and ligand-free route to synthesise size-controlled nanoparticles.^4,21^ Modern systems can create clusters at reasonable rates (on the order of ng per hour) for deposition on flat substrates across the size range from single atoms to a few tens of thousands of atoms, which corresponds to a range of particle diameters from 0.3 nm to ≈8 nm, where the most dramatic particle size effects in catalysis usually take place. The general process of magnetron sputtering and cluster condensation has been widely reported and optimised by some of the authors^4,22,23^ and size-selected clusters of various metals, such as nickel, silver, gold, palladium, *etc.*, have been prepared *via* this route.^21,24–27^ Whilst it remains system dependent, mass resolution of 25 (defined as *M*/Δ*M*), or 4% (Δ*M*/*M*) is readily achievable, which is a particle size variation significantly lower than with most other nanoparticle preparation methods. The lack of solvents during preparation also makes this a potentially much more environmentally friendly catalyst production route, when successfully scaled up.

Our prior studies showed the viability of model catalysts supported by thin alumina films, prepared *via* anodisation of aluminium foil, with Pd deposited *via* a metal evaporator. This showed that the support films were stable to reactive atmospheres with palladium showing redox behaviour in reaction conditions, thus confirming their suitability for use as a model catalyst support.^28^ Small Pd clusters on alumina have previously been studied by Mao *et al.*, who showed significant binding energy shifts over the size range up to 17 atoms.^25^ In their experiments, however, there was no clear relationship between core level shift and particle size. Kaden *et al.* studied Pd clusters between 1 and 25 atoms on titania, and demonstrated that smaller clusters tended to show a greater positive binding energy shift, with a shift of approximately 0.6 eV for Pd_1_.^29^ The same group also observed shifts in binding energy of small Pd clusters as a result of oxygen exposure, typically positive shifts of a few hundred meV.^30^ Schalow and coworkers studied nanoparticulate Pd model catalysts (>1 nm) on Fe_3_O_4_ in a series of ultra-high vacuum experiments and found size-dependent oxidation behaviour with complete oxidation only for smaller clusters and XPS binding energy shifts of up to 1.7 eV for oxidised Pd.^31–33^

Methane is produced from many sources, *e.g.*, compressed natural gas engines produce 9% lower CO_2_ emissions than conventional internal combustion engines, alongside lower particulate and NO_*x*_ emissions, however, due to the emission of unburnt methane, the CO_2_ equivalent (CO_2_e) emissions can be up to 127% higher.^34,35^ To reduce the emission of unburnt methane, hence reducing the CO_2_e emissions, methane oxidation catalysts need to be used in the exhaust.^36^ Typically, metal-oxide-supported platinum-group metal (PGM) catalysts, most commonly palladium, are used for this conversion.^37^ Significant particle size effects have been shown for Pd methane oxidation catalysts previously.^17^ Li *et al.* showed that small Pd clusters (<2.8 nm) encapsulated in a silicalite support were superior to conventionally prepared Pd nanoparticles catalysts with regard to catalyst lifetime.^38^ These studies have, however, not had the precision of control over particle sizes which we are able to demonstrate and have not typically studied the surface composition during the reaction. Deactivation of Pd catalysts in wet reaction conditions, typically with water contents much higher than methane, is a persistent issue, and is directly related to the surface states of palladium.^16,39^ No direct studies exist, however, into how particle size effects this crucial topic.

Near-ambient pressure X-ray photoelectron spectroscopy (NAP-XPS) is a vital technique for understanding surface chemistry of catalysts under reaction conditions. By optimising photon energy to give highly surface sensitive measurements at high resolution, as is only possible at synchrotron light sources, we can study core level shifts, and thus oxidation levels, as a function of the particle size.

## Methods

### Sample preparation

#### Anodization of aluminium

The preparation of alumina films, as used in these experiments, is described elsewhere.^28^ Briefly, strips of aluminium foil (Goodfellow, 99.999%, 25 × 4 mm^2^; thickness 0.50 mm) were cut and cleaned sequentially in ultrasonic baths of ethanol and water for 5 minutes each. Surface reduction was performed electrochemically in 4.7 × 10^−2^ mol L^−1^ sulphuric acid solution. This was followed by a short oxidation step of 30 seconds which produces an approximately 6 nm thick layer of γ-like aluminium oxide covering the aluminium foil. The resulting samples were then again cleaned in ultrasonic baths of ethanol (5 minutes) and then water (5 minutes) prior to cluster deposition and introduced into the deposition chamber of the cluster source.

#### Deposition of palladium clusters

Palladium nanoparticles in the range between 1 and ≈10 000 atoms were produced and deposited onto these thin alumina films using a size-selected dual magnetron cluster source based on the design described in ref. 22 and 23. The palladium target (25.4 mm diameter, 4 mm thickness, 99.999%, ScoTech) was sputtered *via* magnetron sputtering, with a typical power of 10 W. Argon was used as sputtering gas, mixed with helium for condensation at a [Ar] : [He] ratio around 1 : 2; for Pd_1_ pure argon was used. The condensation length, *i.e.* the distance between the face of the target and the iris aperture, which forms the exit of the sputtering chamber, was varied to control the size distribution of particles, as was the iris opening diameter. The clusters were drawn forwards to an ion optics chamber, through a skimmer with an aperture diameter of 1 mm, which was negatively biased to attract the positively charged clusters. The cluster beam was focused through electrostatic lenses onto the entrance of a time-of-flight mass filter, which operated with controlled voltage pulses at specified intervals, to displace the cluster beam and direct only clusters of a specific size (*i.e.* mass-to-charge ratio) into the deposition chamber.^22^ At the start of this study, the mass filter was operated without a “kick plate” which suppresses higher-order (*i.e.* heavier) masses passing through. This lead to a somewhat higher concentration of agglomerated clusters on the samples.

The deposition chamber typically hosted four substrates, two TEM grids and two alumina substrates, along with a blank spot, which allowed for optimisation of the beam prior to deposition. The sample plate was covered by a mask with 4 mm diameter holes over each sample position to ensure only single substrates were deposited on at any time. It was held at negative potential during deposition to attract the positively-charged cluster beam and define the kinetic landing energy of the clusters on the surface. This potential was optimised for each cluster size to ensure the clusters are pinned to the surface and “land” with an energy of less than 1 eV per atom to prevent post-deposition agglomeration caused by migration and destruction on the surface.^40^ The beam current was measured by a current amplifier connected to the sample plate. The most important deposition parameters are listed in the SI (Table SI 1). Once a stable current was reached, the deposition time was calculated (based on the integral of cluster current and deposition time) such that a particle coverage (defined as ratio between area covered by the clusters and total surface area) between 1% and 2% was achieved. For some experiments, the sample stage was manually rastered to ensure a more homogeneous cluster distribution over the substrate and avoid agglomeration in certain areas.

In all cases, clusters were also deposited onto TEM grids (EMResolutions, carbon film on copper 300 mesh) under identical conditions, either before or after the deposition onto alumina, to provide a reference sample for particle size analysis. The range of particle sizes created, see Table 1, reflects the sizes of nanoparticles on industrially relevant catalysts for methane oxidation.^17^ No attempt was made to produce particles with more than 10 000 atoms in order to ensure rapid deposition (<1 hour) whilst retaining a 1–2% surface coverage, which in turn enables high-resolution NAP-XPS measurements on a reasonable time scale (<1 hour for a Pd 3d core level spectrum). The samples were transferred through air to the respective instruments for TEM and XPS measurements.

**Table 1:** Pd clusters produced in this experiment, where Pd_*x*_ refers to a palladium cluster containing *x* atoms. Diameters are approximated, assuming spherical nanoparticles. NAP-XPS performed in multiple gas conditions for catalysts listed with “Y”. TEM images collected for various samples with “Y”. The sputtering power, deposition current and total time are tabulated in the SI for each sample produced. Pd 3d_5/2_ binding energies and shifts relative to Pd foil for all clusters, measured in vacuum with photon energy 890 eV except where noted

Cluster	Theor. Diameter/nm	NAP-XPS	TEM	Pd 3d_5/2_ BE/eV	BE shift/eV	Notes
Pd_1_	0.28	N	N	336.82	+1.65	Photon energy 870 eV; exit slit 25 µm
Pd_13_	0.71	Y	N	335.87	+0.70	—
Pd_55_	1.16	Y	Y*	336.24	+0.97	Exit slit 25 µm; slight P contamination
Pd_100_	1.41	N	Y*	—	—	No XPS
Pd_147_	1.60	Y	Y*	335.85	+0.68	—
Pd_509_	2.43	Y	N	335.74	+0.57	—
Pd_1000_	3.04	N	Y	—	—	No XPS
Pd_1250_	3.27	Y	N	335.41	+0.14	—
Pd_2500_	4.13	Y	N	335.73	+0.46	—
Pd_10000_	6.55	Y	N	335.47	+0.30	—
Pd foil	∞	—	—	335.17	±0.00	Reference

Pd_100_ and Pd_1000_ were test samples to ensure differences could be seen under vacuum conditions, and were not subjected to NAP-XPS studies. NAP-XPS measurements in the Pd 3d region of a Pd_1_ sample were attempted, but deemed to not be viable for a full study as it required more than four hours per scan at suitable resolution.

#### Particle size characterisation by TEM

Two instruments were used for Transmission Electron Microscopy (TEM). Instrument E01 (JEOL ARM200F) at the electron Physical Science Imaging Centre (ePSIC) at Diamond Light Source allows high Resolution STEM aberration corrected imaging at 200 kV and 80 kV, in High-Angle Annular Dark-Field (HAADF), Low-Angle Annular Dark-Field (LAADF), Bright-Field (BF) and Annular Bright-Field (ABF) modes.^41^ The JEM-2100 microscope at the Research Complex at Harwell (RCaH) operates at 200 kV and offers various illumination conditions including standard non-aberration corrected parallel and convergent beam, and scanning TEM capability.^42^

The cluster size distribution for each sample was determined using intensity analysis on high and low magnification HAADF-STEM images in ImageJ. Clusters were identified on the basis of their pixel intensity after the background carbon intensity was subtracted. The mean diameter was determined by averaging multiple diameter measurements around equivalent circles. The number *N* of atoms in the cluster was estimated assuming spherical particles, according the following equation1
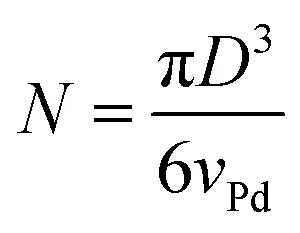
where *D* is the particle diameter and *v*_Pd_ = 0.0147 nm^3^, the volume occupied by a single atom in the palladium fcc lattice.

## Synchrotron experiments

### XPS experiments

All XPS measurements were performed at the VerSoX beamline B07 of Diamond Light Source, using the “TPOT” endstation, which is equipped with a 150 mm hemispherical electron energy analyser (SPECS PHOIBOS). The general operation of this beamline has been described previously.^43^ For most measurements a fixed photon energy of 890 eV was used. Combining the 400 L mm^−1^ monochromator grating, an exit slit of 37.5 µm, and an analyser pass energy of 20 eV, an overall resolution of 0.37 eV was achieved. In the limited cases where other photon energies were used, these are mentioned in the text.

Gases were dosed using the B07 gas system, with the exception of water which was introduced through a manual leak valve. Gas ratios for [CH_4_] : [O_2_] : [H_2_O] of 1 : 30 : 0 and 1 : 30 : 30 were used as the “dry” and “wet” methane oxidation conditions with total pressures of 4 mbar and 8 mbar, respectively. Experiments were run in a low flow mode, with the gas only pumped through the small apertures of the differentially pumped analyser and the beamline entrance, which each have a diameter of 0.3 mm.^43^ Al 2p and Pd 3d core level spectra were recorded at 50 °C intervals from 150 °C to 450 °C in each gas condition. A mass spectrometer located in the differential pumping system of the electron analyser allowed monitoring the reaction products.

### XPS data analysis

In order to compensate for charging-related energy shifts and beamline/analyser energy offsets, Al 2p spectra were measured interlaced with Pd 3d, and the binding energy (BE) axes of all spectra were calibrated with respect to the main Al 2p peak of Al_2_O_3_ at BE 74.6 eV. In some cases, small variations in peak positions (typically <0.2 eV) were observed in vacuum at room temperature over the data acquisition time of approximately 1 hour. As the corresponding Al 2p spectra confirmed that these were charging shifts of the entire sample, the Pd 3d spectra were manually aligned over separate iterations. An example of this is shown in the SI (Fig. SI 2).

Normalisation was applied at the low binding-energy side of each spectrum and a Shirley background^44^ was subtracted from all Pd 3d spectra shown here. The spectra were fitted with 4 peaks, reflecting the metallic (Pd/Pd(0)) and oxide (PdO/Pd(ii)) species in the 3d_5/2_ and 3d_3/2_ spin–orbit split components. Based on our prior experience, and literature data,^16,39^ there was no need for fitting of any additional, higher binding energy species to reflect other palladium states for these samples. Positions for each component were allowed to vary slightly across different samples and different temperatures for the same sample as core level shifts change with particle size and overall oxygen content.^45^ Typically the binding energy difference between the two chemical states is 1.4–1.6 eV.

## Results and discussion

### TEM

The successful production of size-selected clusters was confirmed for various depositions by recording TEM images from clusters deposited onto carbon grids. These samples were prepared immediately after the depositions onto alumina, and as such are expected to be comparable.

**Fig. 1:**
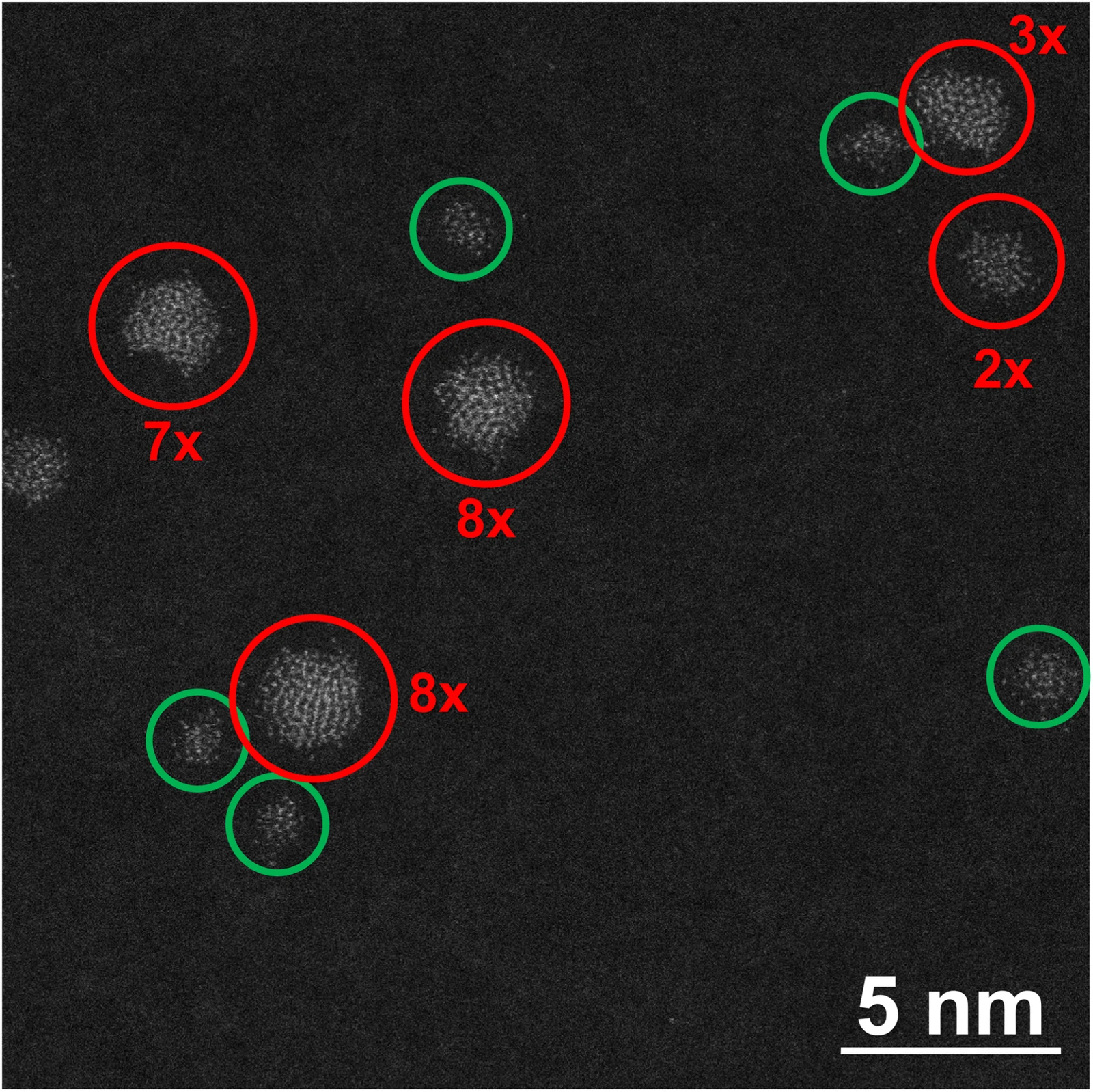
TEM image of Pd_55_ clusters, highlighting correctly sized clusters (green circles) and some larger agglomerates (red circles, approximate number of atoms shown as factor of Pd_55_).

**Fig. 2:**
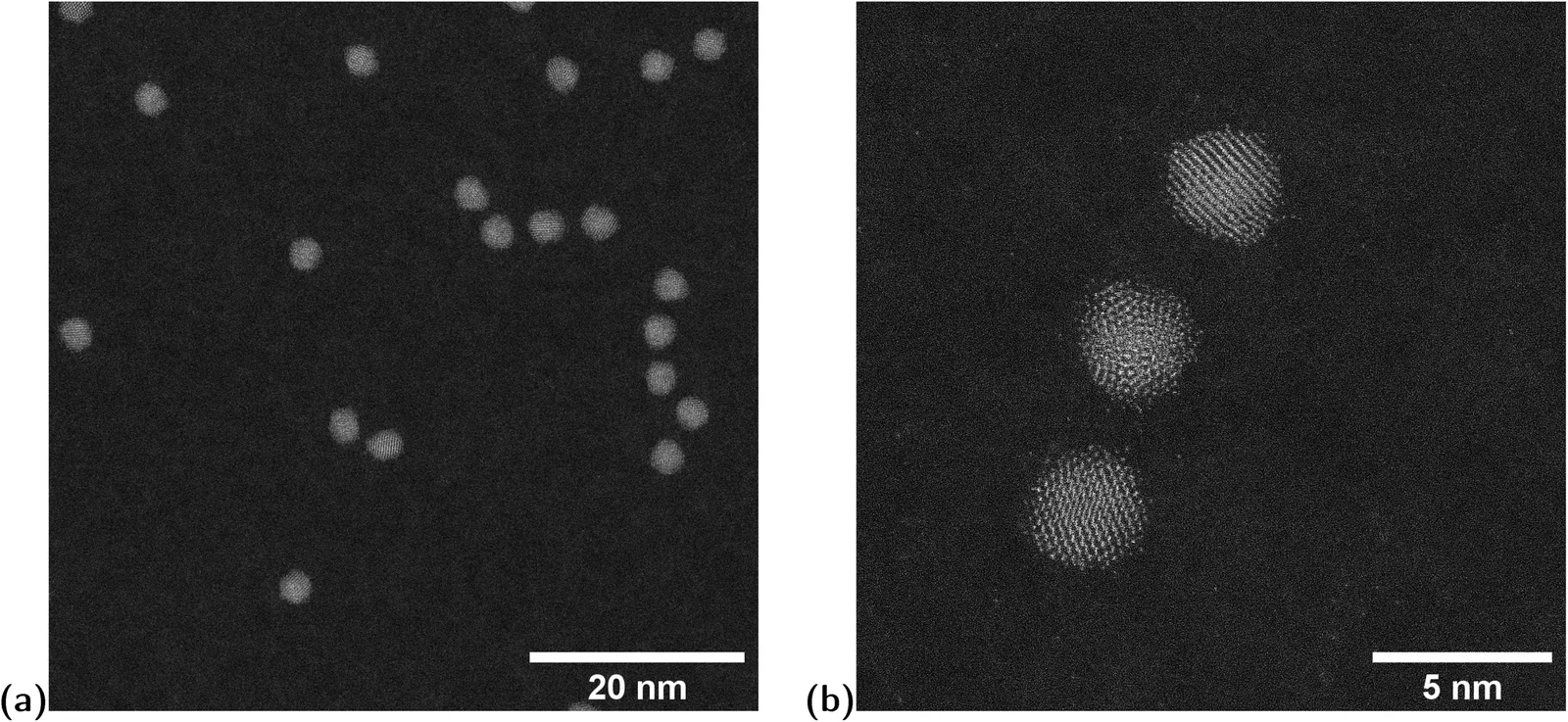
TEM images of Pd_1000_ clusters, taken at low (a) and high (b) magnification.

**Fig. 3:**
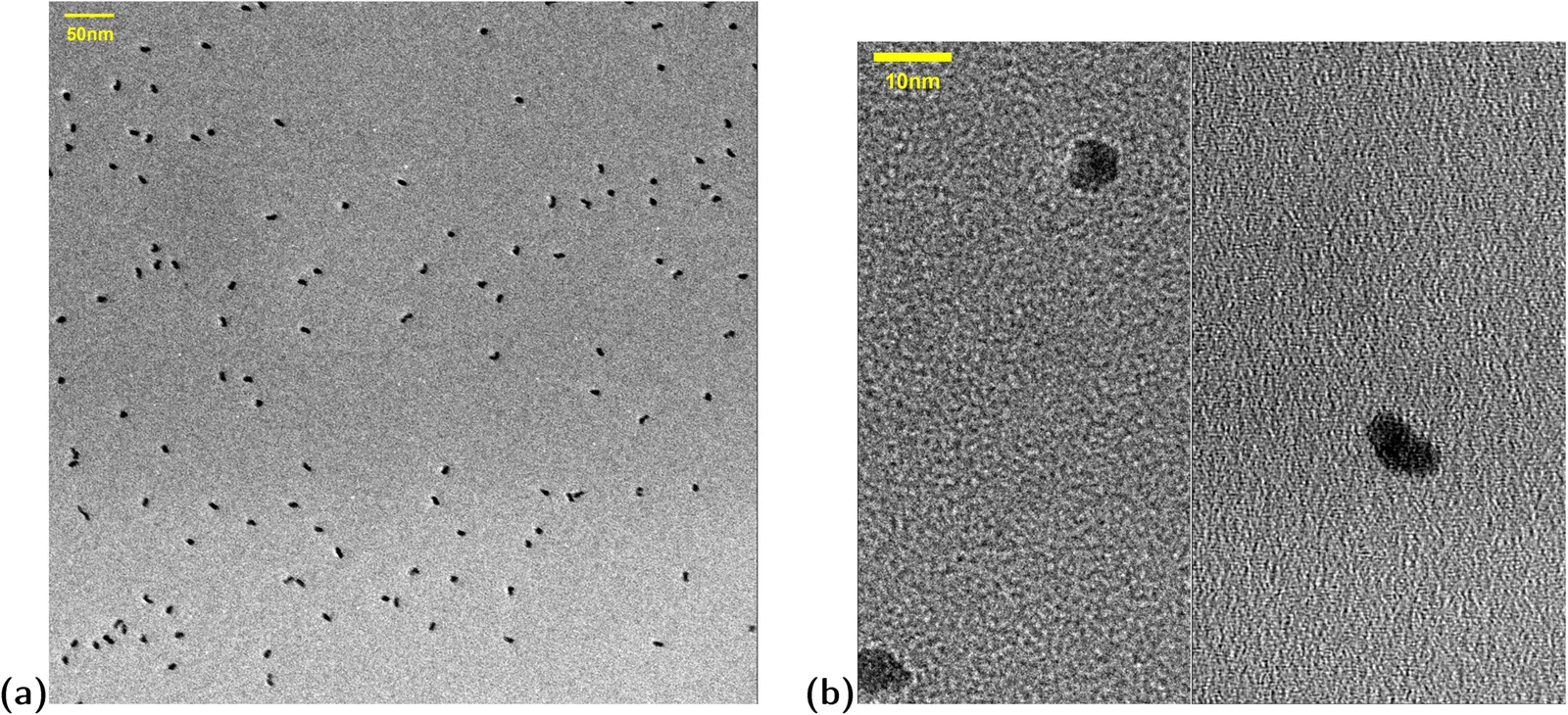
TEM images of Pd_10000_ clusters, taken at low (a) and high (b) magnification.

Pd_55_ clusters are shown in Fig. 1. This image, recorded on an aberration-corrected (AC) STEM, clearly shows individual atoms and internal cluster structure. It is clear that a significant number of larger clusters are present in addition to the nominal 55 atom clusters. The possibility that larger clusters could pass through the mass filter is unlikely as filtering is performed within a 4% tolerance and higher orders are eliminated by using an additional kick-plate in the mass filter. Clusters could, however, agglomerate on the carbon substrate, due to the relatively high mobility on carbon and the high density of deposition. There is also the less likely option that particles collide and agglomerate in the region after the filter and before deposition. The region where this is possible is around 100 mm, but all clusters which have passed through the filter should have the same charge and thus repel each other, making combination less likely. Pd_1000_ clusters were also studied using AC-STEM, and the resulting images are shown in Fig. 2. The low magnification images (such as in Fig. 2a) show minimal agglomeration into particles of significantly more than 1000 atoms. The high magnification images (Fig. 2b) allowed for accurate size determination, leading to an average of 1003 ± 40 Pd atoms per cluster. Pd_10000_ clusters on carbon grids were imaged using a standard, non-aberration corrected TEM (JEM-2100), resulting in lower resolution images, as shown in Fig. 3. In the magnified image, Fig. 3b, a mix of single and paired clusters is clearly present. Low magnification images show, however, that there are very few excessively large clusters, as opposed to Pd_55_ but similar to Pd_1000_. This is likely to be a result of the lower mobility and the higher stability of the larger particles. The cluster beam current achieved for Pd_10000_ was approximately 10 times lower than that typically measured for clusters between 13 and 2500 atoms. This observation suggests that the probability of cluster growth to this size is an order of magnitude lower than for smaller sizes, even with the gas pressure and other source parameters optimised for maximum cluster current for each size. For this reason, no attempt was made to produce clusters greater than 10 000 atoms.

### XPS in vacuum

**Fig. 4:**
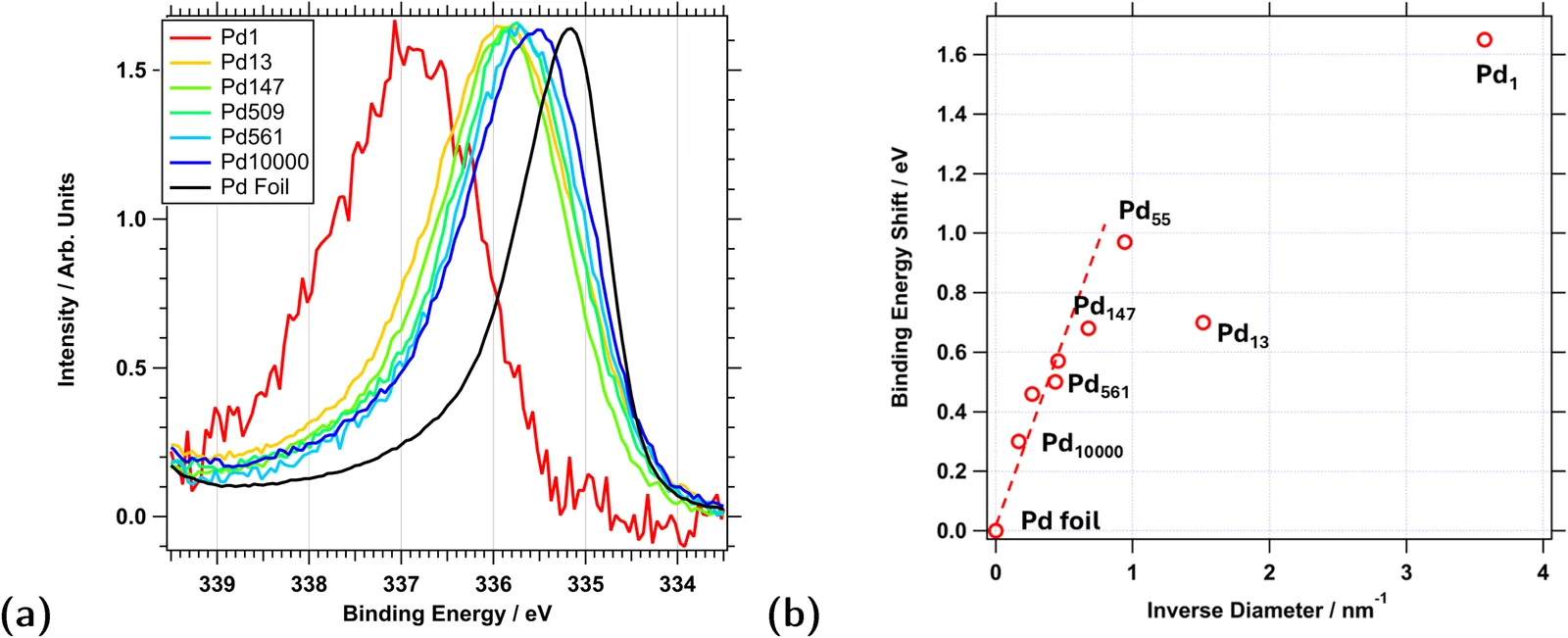
(a) Pd 3d_5/2_ region UHV XPS scans for various sizes of Pd clusters on alumina film, along with Pd foil as a reference. Peak intensities are normalised to 1 in all cases to make shifts clearer. (b) Plot of Pd 3d binding energy shift as a function the inverse cluster diameter.

High-resolution XPS measurements were preformed in vacuum on all samples prior to exposure to reactive gas mixtures. They show clear shifts in the Pd 3d_5/2_ binding energy as a function of particle size, as can be seen in Fig. 4a. For comparison, a reference spectrum of a palladium foil, recorded with similar beamline and analyser parameters, is also included in the figure. In the latter spectrum, the Pd 3d_5/2_ peak appears at BE 335.17 eV, whereas all cluster spectra are shifted towards higher binding energies.

A list of all measured binding energies is presented in Table 1. During these vacuum experiments it was observed that contaminants (*e.g.* C or P) had a significant effect on the Pd 3d binding energy shifts. Contamination was therefore carefully monitored and only clean surface data were used for the analysis of this data set and Fig. 4.

As a general rule, with the exception of Pd_13_, the smaller the particle the larger the positive binding energy shift when compared to the foil reference. Pd_1_, the single atom sample, showed a shift of +1.65 eV, whilst Pd_10000_, the largest clusters studied here, shifted by only +0.30 eV. Except for Pd_1_, the cluster size does not seem to affect the Pd 3d peak width. A slight increase is observed for the Pd_13_ clusters, but the same increase is also seen in the corresponding Al 2p spectrum and therefore likely due to inhomogeneous charging of the support, see Fig. SI 3 of the SI.

Between approximately Pd_147_ and Pd_10000_, the shift follows a linear trend as a function of the inverse diameter (see Fig. 4b), which also includes the data point for the Pd foil and is in agreement with earlier observations by Wertheim and DiCenzo,^46^ who explained these shifts with final state effects due to the positive charge of the electron hole distributed over the entire surface of a spherical particle. Clearly, this assumption breaks down for very small particles, where the shifts are dominated by surface effects and support interaction.

For Pd_1_, all Pd atoms are at the surface and in contact with the support. Agglomeration into larger particles must be expected to happen for a significant fraction of atoms at the experiment temperature of 150 °C. This is most likely the reason for the large observed width of the Pd 3d_5/2_ peak. For Pd_13_, all but one Pd atom (92%) are either exposed to vacuum or the support (assuming a fcc-like structure). Consequently, interface effects dominate the binding energy shifts of these clusters.^25^ For Pd_55_ and Pd_147_, the fractions are 76% and 62%, respectively, and for Pd_10000_ it is estimated around 15%. For the larger particles, the attenuation of photoelectrons also needs to be considered as the cluster size already significantly exceeds their inelastic mean free path (IMFP) of 0.86 nm for the kinetic energy 550 eV used in this study, therefore only bulk and surface atoms but no interface atoms contribute to the signal. For Pd_10000_ the fraction of surface atoms contributing to the XPS signal is estimated to be around 5%. These variations in the contributions of surface and interface atoms and the variation in the chemical environments of these atoms explain, why no universal trend is observed in binding energy shifts as a function of particle size.

### NAP-XPS under reaction conditions

**Fig. 5:**
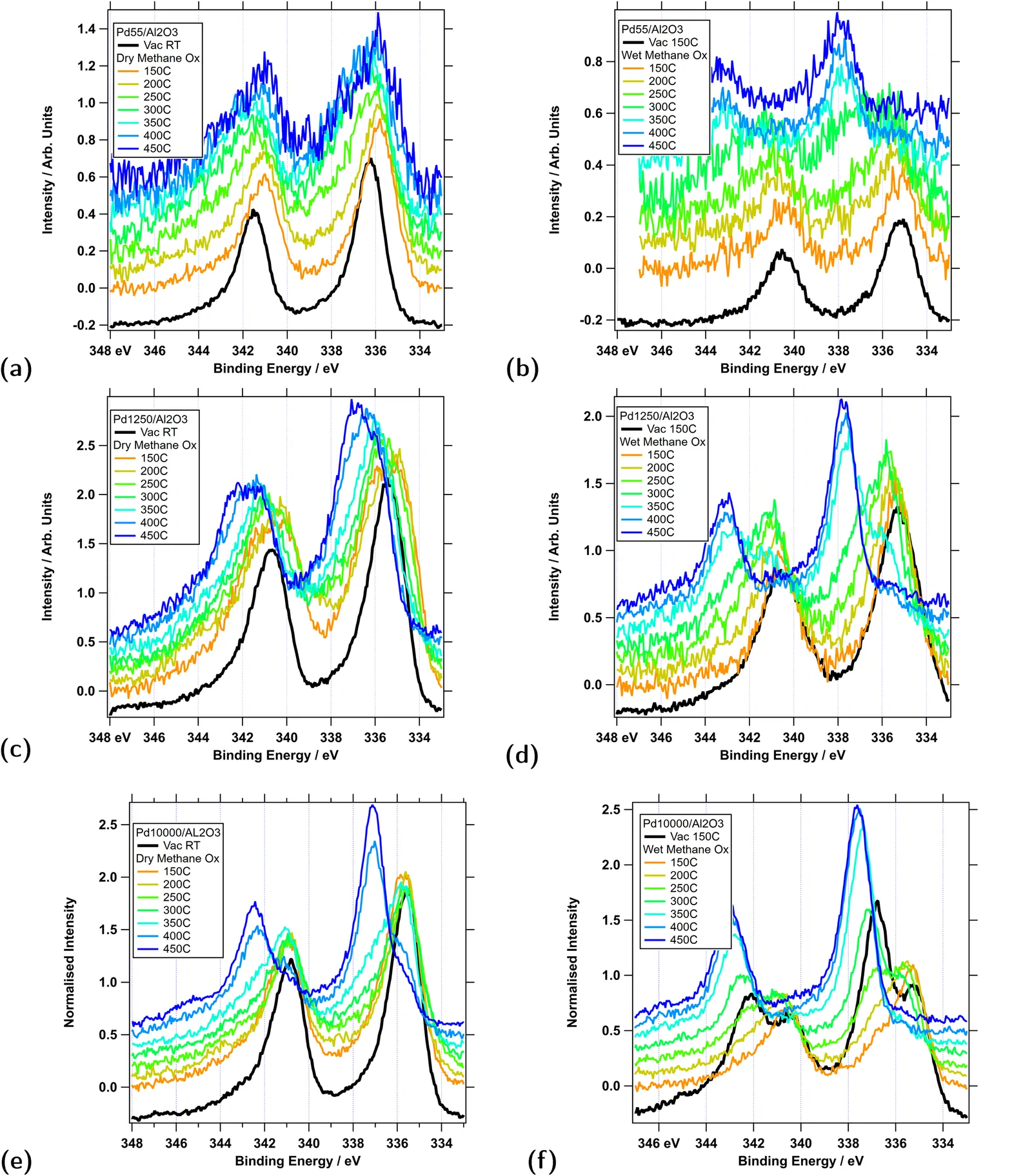
Series of NAP-XPS spectra for different temperatures under dry and wet methane oxidation conditions (see text for more details): (a) Pd_55_ dry; (b) Pd_55_ wet; (c) Pd_1250_ dry; (d) Pd_1250_ wet; (e) Pd_10000_ dry; (f) Pd_10000_ wet. Photon energy 890 eV; the spectra are offset for better clarity.

NAP-XPS measurements were performed to characterise the chemical changes Pd catalysts of different sizes undergo during the methane oxidation reaction. Fig. 5 shows Pd 3d spectra for three particle sizes, Pd_55_, Pd_1250_, and Pd_10000_, recorded for different temperatures under dry and wet methane oxidation conditions. All samples for this series were deposited without rastering (“single spot”). Each sample was introduced into the endstation from air, annealed to 150 °C, and a series of reference spectra was measured in vacuum at this temperature. The sample was then exposed to dry methane oxidation conditions ([CH_4_] : [O_2_] : [H_2_O] = 1 : 30 : 0; 4 mbar) and annealed to 450 °C in steps of 50 °C. At each temperature a series of survey, Pd 3d, Al 2p, C 1s, and O 1s XPS spectra were recorded. The corresponding Pd 3d spectra are shown in Fig. 5a, c and e; the other spectra were mainly for binding energy calibration and checking contamination and are not shown. Once the 450 °C measurements were complete, the endstation was evacuated, the sample was left at 450 °C for about 10 min, and then slowly cooled down to 150 °C. A second series of reference spectra was measured in vacuum at 150 °C. These spectra are included in Fig. 5b, d and f (black lines) and show fully reduced Pd particles, with the exception of Pd_10000_. Following this, the sample was exposed to wet methane oxidation conditions ([CH_4_] : [O_2_] : [H_2_O] = 1 : 30 : 30; 8 mbar) and the annealing steps were repeated.

All series of spectra clearly show an evolution from metallic Pd(0) with a single Pd 3d_5/2_ peak around BE 335.9 ± 0.4 eV to mixed Pd(0) and Pd(ii) compositions with an additional peak around BE 337.1 ± 0.4 eV. The binding energy shifts upon oxidation are in line with findings reported previously for Pd clusters of similar sizes.^25,29–31^ The concept of assigning oxidation states to individual atoms in supported small clusters has, however, been drawn into question in a recent publication by Perco *et al.*. Through a combination of XPS measurements and DFT modelling these authors found that core level binding energies of the metal atoms in Mo and W clusters can vary by more than 1 eV for atoms which should nominally be considered in the same oxidation state based on their coordination and/or Pauling valency.^45^ We therefore refrain from fitting the spectra with peaks at fixed binding energies corresponding to certain oxidation states and, instead, use a two-state model with peaks of variable binding energy, separated by approximately 1.5 eV (±0.1 eV), which are assigned to metallic and oxidised Pd. The latter is at the same binding energy as Pd(ii) for the large clusters. Therefore we refer to this state as Pd(ii) for the rest of this discussion.

**Fig. 6:**
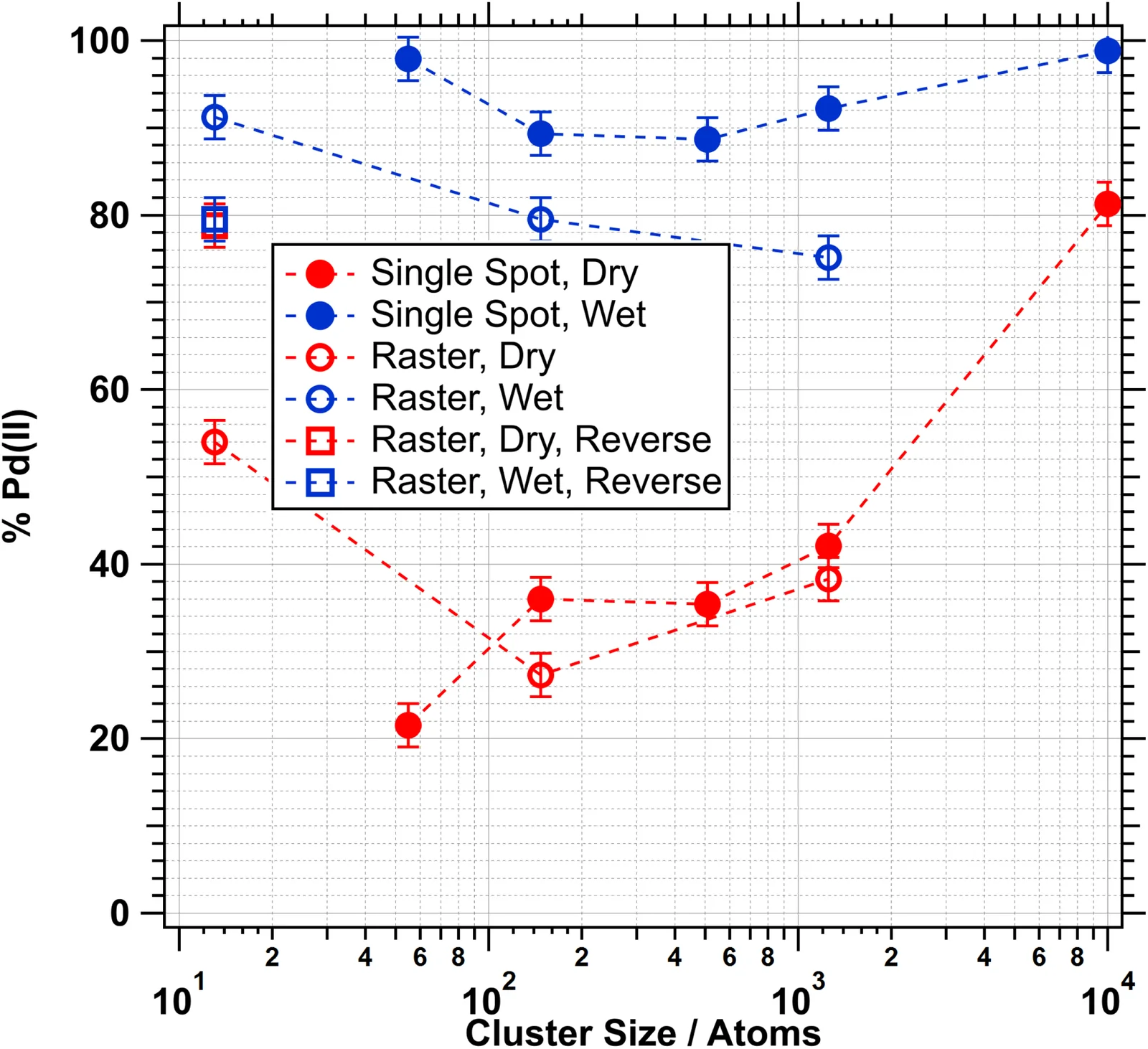
Plot of percentage of oxidised Pd(ii), as determined by XPS fitting, for various clusters. The different symbols represent single spot and rastered depositions under dry and wet conditions. Each percentage represents the maximum oxidation level reached during the ramp, typically at 450 °C. Error bars represent a variation of 5%, the estimated error in the fitting procedure.

For dry methane oxidation (Fig. 5a, c and e) broadening towards the Pd(ii) signal typically first appears when the temperature is raised to above 300 °C. There are, however, pronounced differences between catalysts of different sizes. The degree of oxidation, *i.e.* the fraction of the Pd(ii) peak compared to the entire peak area, increases with particle size. At the final temperature, Pd_55_ is to 22% oxidised, Pd_1250_ 42% and Pd_10000_ 81%. A graphical representation of the final oxidation levels, as determined by XPS fitting for all particle sizes, is presented in Fig. 6 (red filled symbols). This shows a broad range of particle sizes, between 147 and 1250, for which the oxidation level is around 40%; for the largest particles, Pd_10000_, it is significantly higher, and for the smaller clusters Pd_13_ and Pd_55_ the levels oscillate. Smaller particles generally reach their maximum oxidation levels at lower temperatures and more gradually than larger particles. For Pd_10000_ large levels of oxidation are only seen after annealing to 400 °C and the data seem to indicate that full oxidation could have been achieved for higher temperatures. However it also needs to be considered that the onset of reduction generally starts at around 500 °C and samples could not be heated higher as the Al_2_O_3_ film starts to decompose.

The picture is very different for wet methane oxidation conditions, see Fig. 5b, d and f. For all Pd particle sizes, compared to dry conditions, oxidation starts at lower temperature, typically between 200 °C and 250 °C, and the oxidation level at 450 °C is higher, typically above 90%, as shown in Fig. 6 (blue filled symbols).

Given the order of experiments, with the wet annealing series carried out after the dry experiments, we cannot exclude that a degree of sintering or agglomeration of particles has happened during the fist (dry) annealing step. In order to address this question, a second series of experiments was carried out with samples, where the particles were deposited with a lower density (coverage 1% instead of 2% and while slowly moving the support (“Raster”, see SI for more detail), but otherwise adhering to the same protocol as outlined above. The lower density and more homogeneous distribution of particles should reduce the likelihood of agglomeration. In all experiments with rastered samples we find slightly lower levels of oxidation at the highest temperature, see Fig. 6 (open symbols), both for dry and wet conditions. But the general trend remains that wet conditions lead to higher levels of oxidation than dry conditions.

**Fig. 7:**
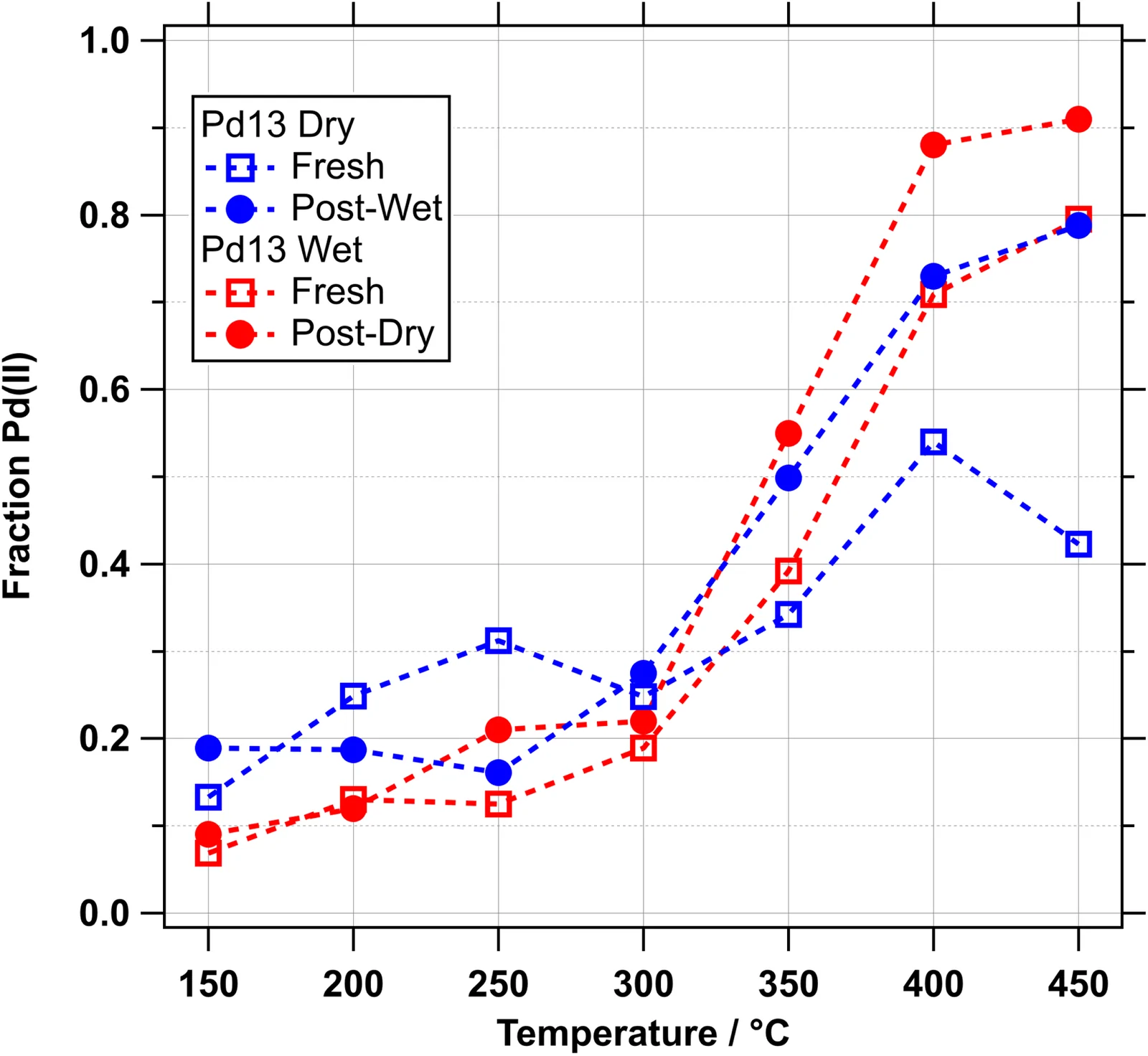
Graph showing variation in Pd(ii) percentage (as part of total Pd 3d peak area) over NAP-XPS experiments performed on Pd_13_ cluster samples. See text for detail.

Reversing the order of conditions was only tested for the smallest cluster size, Pd_13_, as the effect of agglomeration is likely to be most significant for small clusters. Two nominally identical Pd_13_ samples were prepared by raster deposition. Fig. 7 shows the changes in oxidation levels for one sample being exposed to methane oxidation conditions in the standard order, *i.e.* vacuum-dry-vacuum-wet (experiment 1; blue open squares and red filled circles), and the other in reverse order, vacuum-wet-vacuum-dry (experiment 2; red open squares and blue filled circles). Experiment 1 shows the same trend as observed for all other sample sizes, *i.e.* higher oxidation levels for the “Post-Dry” wet conditions compared to the “Fresh” dry conditions. Experiment 2 shows more or less the same behaviour for the “Fresh” sample in wet conditions, as observed in experiment 1 for the “Post-Dry” wet conditions, albeit at somewhat lower oxidation levels, typically 10 percentage points below experiment 1. The big difference is in the dry series that follows the wet annealing in experiment 2. This follows almost the same path as the previous series under wet conditions. We must conclude that the wet methane oxidation conditions lead to irreversible changes in the particles. These changes are most likely due to sintering or agglomeration of particles, as the sample can be almost fully reduced after the fist annealing step of experiment 2, which can be seen by comparing the first data points of each annealing series in Fig. 7. Surface hydroxyl, either on the alumina support or on the Pd clusters could enhance the mobility of particles and/or single atoms at elevated temperatures and thus favour the formation of larger clusters, which are more easily oxidised.

**Fig. 8:**
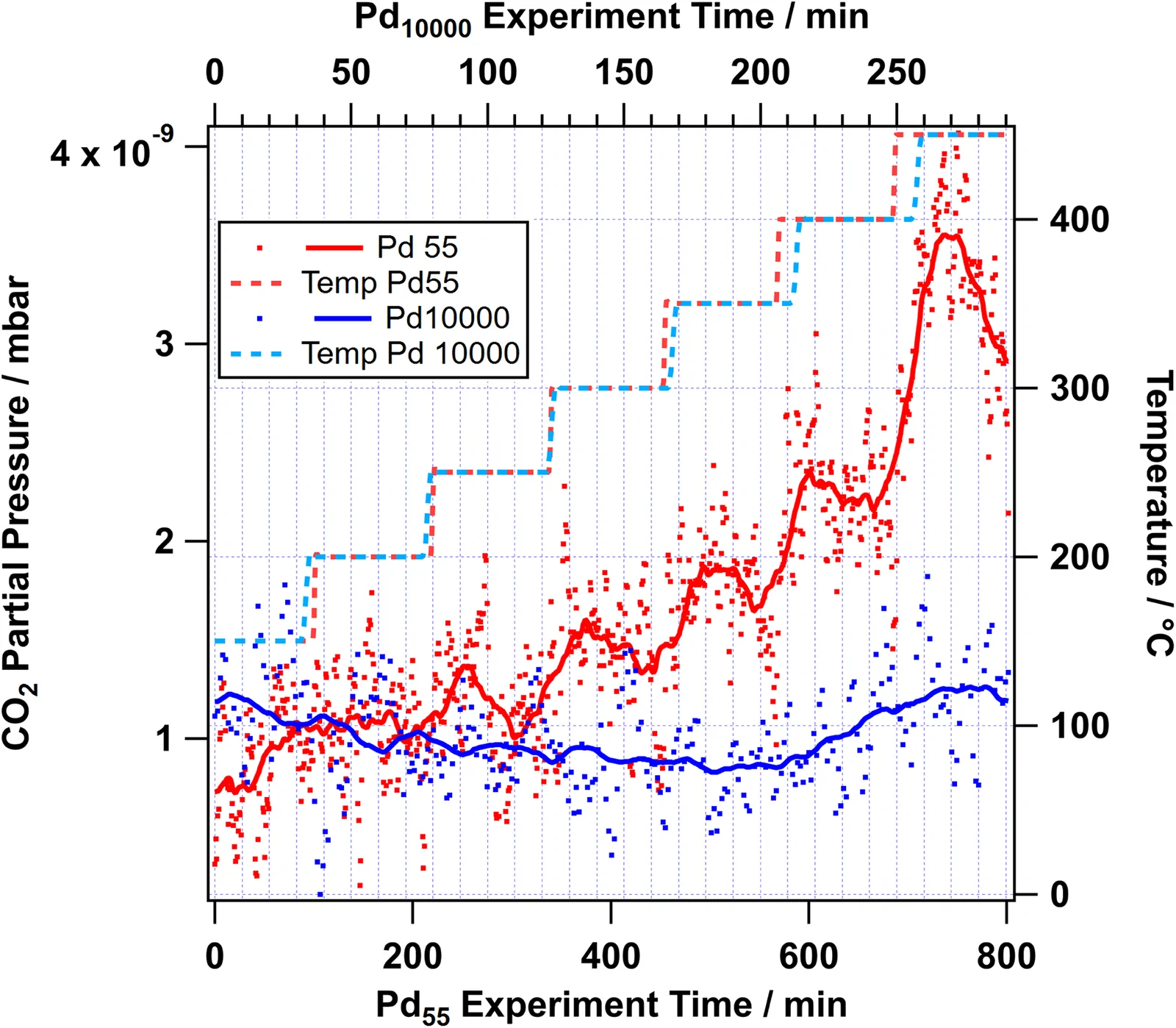
RGA data recorded for Pd_55_ and Pd_10000_ samples during dry NAP-XPS experiments. Dots represent individual data points, solid lines represent data smoothed over a 10 min window.

Gas analysis was performed simultaneously with the NAP-XPS experiments using a mass spectrometer located in the first pumping stage of the electron analyser, which detects primarily gas in the vicinity of the sample.^43^ Detecting reaction products for these samples is difficult, as the active catalyst area is only of the order of 0.1 mm^2^, *i.e.* about 10^−5^ to 10^−4^ that of a typical powder catalyst studied under similar conditions (typically 10^−3^ to 10^−2^ m^2^ for 1 mg of supported catalyst). Therefore, significant CO_2_ yields could only be detected for the most active catalysts. The CO_2_ yield of Pd_55_, which was most active under dry methane oxidation conditions, is shown as an example in Fig. 8. Despite the high noise levels, the data show a clear increase in the CO_2_ signal (mass 44) as a function of temperature, between 350 °C and 450 °C. This temperature range is about 100 °C higher than the light-off measured for supported Pd/Al_2_O_3_ powder catalysts.^16^ Given that the flow rate in our NAP experiments (0.5 sccm methane) is much lower than in a typical reactor experiments, this difference is not surprising. The data for the Pd_10000_ sample, also included in Fig. 8, only show a very small increase in the CO_2_ signal above 400 °C. Given that the active area of both samples should be approximately the same, this indicates lower activity for the larger catalyst particles. These findings are in agreement with our earlier work, which indicates that a mix of Pd(0) and Pd(ii) needs to be present at the surface for high complete methane oxidation yields.^16,17,39^ A clear trend could, however, not be detected due to the overall very low signal levels.

## Conclusions

Palladium clusters over a wide size range of 1 to 10 000 atoms were deposited onto a flat Al_2_O_3_ support, using a size-selected dual magnetron cluster source, and studied by vacuum and NAP-XPS. In vacuum, significant core-level shifts, up to 1.65 eV, were observed as a function of size, with the smallest clusters shifting to the highest binding energies. Under industrially relevant methane-oxidation gas conditions, clusters of all sizes undergo oxidation, with the extent and temperature-dependence of this oxidation varying with cluster size. Under dry conditions ([CH_4_] : [O_2_] : [H_2_O] = 1 : 30 : 0; 4 mbar) the largest clusters (Pd_10000_) were oxidised almost completely upon heating to 450 °C, while smaller clusters (≤1250 atoms) typically showed a mix of Pd(0) and Pd(ii), with the latter around 30% of the total XPS signal. Under wet methane oxidation conditions ([CH_4_] : [O_2_] : [H_2_O] = 1 : 30 : 30; 8 mbar) oxidation occurs more readily and reaches around 80–100% for all particle sizes. Wet conditions also appear to favour significant sintering and/or agglomeration, especially of smaller particles. This leads to much higher levels of oxidation under dry conditions when the sample has been exposed to wet conditions before. Low levels of gas conversion, near the detection limit, can be measured directly by mass spectrometry, with Pd_55_ showing the highest activity of all measured samples.

This study highlights the capabilities of NAP-XPS in combination with size-selected cluster deposition for studying particle-size effects. Remaining challenges are a more detailed understanding of the observed Pd 3d core-level shifts in terms of oxidation *vs.* support interaction, which has to involve DFT modelling, and a better characterisation of the sintering/agglomeration effects during annealing. This is part of ongoing work.

## Author contributions

Conceptualization: A. I. L., G. H., R. E. P; writing – original draft: A. I. L., G. H.; writing – review & editing: H. H., J. J. C. C., M. A. V. S., S. K., D. C. G., P. F., R. E. P.; data curation: A. I. L., H. H., J. J. C. C., M. A. V. S., S. K., D. C. G., P. F.; formal analysis: A. I. L.; funding acquisition: G. H., R. E. P.; methodology: H. S., E. J., H. H., B. V. I.; supervision: G. H., R. E. P.

## Conflicts of interest

There are no conflicts to declare.

## Supplementary Material

FD-267-D5FD00171D-s001

## Data Availability

Raw data are accessible (https://doi.org/10.5281/zenodo.17954331). *Operando* XPS studies of precisely size-selected Pd nano-catalysts for methane oxidation – raw data used in graphs (https://zenodo.org/records/17954332). Supplementary information (SI) is available. See DOI: https://doi.org/10.1039/d5fd00171d.
