# Supplementary material for: Operando XPS studies of precisely size-selected Pd nano-catalysts for methane oxidation
Source: Faraday Discuss. 2026 Mar 18;267:521–38. doi: 10.1039/d5fd00171d (PMC13576673; doi:10.1039/d5fd00171d)
Supplement: Supplementary file 1 [file FD-267-D5FD00171D-s001.pdf]

Supplementary Information (SI) for Faraday Discussions.
This journal is © The Royal Society of Chemistry 2026

Supporting Information:

Operando XPS studies of precisely size-selected Pd nano-catalysts for methane oxidation

Alexander I. Large,[a] Henry Hoddinott,[a,b] Haamidah Sana,[a] Elizabeth Jones,[c] James J. C. Counter,[a,d] Matthijs A. van Spronsen,[a] Santosh Kumar,[a] David C. Grinter,[a] Pilar Ferrer,[a] Bernd von Issendorf,[e] Richard E. Palmer,[b] and Georg Held[a *]

[a] Diamond Light Source, Didcot, UK
[b] Swansea University, UK
[c] Oxford University, UK
[d] University of Reading, UK
[e] Albert-Ludwigs-Universität, Freiburg im Breisgau, Germany

∗ Diamond Light Source, Harwell Science Campus, Didcot OX11 0DE, UK;
Email: georg.held@diamond.ac.uk

Contents

Cluster Deposition 2
Parameters 2
Single Spot vs Rastering 3
XPS Data Analysis 4
XPS Pd3d region data correction 4
Results 5
Al 2p vs Cluster size 5
Contamination 6

Cluster Deposition

Parameters

Sample	Sputtering Power / W	Average Dep Current / pA	Deposition Time / s	Average Surface Loading %
Pd1	9.61	348	1500	1.88%
Pd13	10.02	13	4200	1.09%
Pd55	10.24	15	2535	1.98%
Pd147 v1	10.05	28	714	2.01%
Pd147 v2	9.99	16	660	1.06%
Pd509	9.96	32	380	2.79%
Pd561	10.09	21	210	1.08%
Pd1250 v1	9.96	11	435	2.00%
Pd1250 v2	10.09	11.5	210	1.01%
Pd2500	9.96	23	130	1.99%
Pd10000	10.06	2	600	2.01%

Table SI 1: Details of depositions for Pd clusters. Samples with ~1% loading were rastered during the deposition process. All other samples were left static during deposition, with a total coverage of approx. 2%.

For Pd_{13}, the sample was cut into two pieces after deposition, to enable two independent experiments on clusters of the same size. For Pd_{2500}, the sample was measured initially in only vacuum (v1) then stored until a later experiment (v2). Cluster production was conducted in two waves: the 2% loading, static depositions ("Single Spot") followed by the 1% loading, rastered depositions. Surface loadings are estimated as total surface site coverage, based on expected particle diameters of spherical clusters.

Deposition currents were typically in the range 10-30 pA. The only major outliers are the single atom Pd_1 sample (348 pA) and the largest sample, Pd_{10000} (2 pA). This suggests that the rate of cluster deposition for single atoms is typically on the order of ten times faster than for typical mid-size clusters, which is itself ten times faster than for the largest clusters. Within the Pd_{13}-Pd_{2500} range, there is no clear trend to the achieved current. It is worth noting that whilst sputtering power remains a near-constant throughout (typically 10 W, varying between 9.96 W and 10.09 W for all but two samples), other parameters on the source were also varied to achieve optimal deposition currents. These include the iris opening at the end of the source chamber and the biases across various optical elements. Condensation length was typically kept the same within waves of experiments, with the first wave using a length of 140 mm, whilst 230 mm was used for the second wave. Reducing the condensation length reduces the probability of large cluster formation and can also produce more non-equilibrium structures of clusters than a larger condensation length. It is worth noting that where individual waves of experiments sought to produce clusters of the same size, variation of parameters was necessary to optimise the cluster current. This could be due to a range of factors including, but not limited to: target degradation, chamber temperature variation, and chamber base pressure/contamination. Throughout these experiments, the same Pd target (25.4 mm diameter, 4 mm thick, 99.99% purity, ScoTech) was used. As such, after the first depositions, a ring had formed in the surface, where atoms had been sputtered away. Aside from requiring varied source parameters, it is not anticipated that this has a significant effect on the experiments presented.

Single Spot vs Rastering

Initial sample preparation consisted of depositing clusters in a single spot on the alumina substrates. This lead to significant variability in the effective Pd : Al ratio across the samples. As such, the first stage of any analyses was trying to find a representative spot on a sample, to keep the Pd :Al ratios as similar as possible across the size range.

In an attempt to, both avoid this and see if it had significant impacts on the resulting data, the second wave of clusters were produced using rastering, where clusters were deposited across the samples rather than in a single spot. The total time and cluster coverage was maintained, theoretically resulting in a lower maximum coverage (1% instead of 2%), but a more consistent average. Two different cluster sizes across the range were selected, Pd_{147} and Pd_{1250}. Vacuum measurements of Pd_{147} gave a difference of only 0.01 eV in the Pd $3d_{5/2}$ peak position, as shown in Fig. SI 1, which demonstrates the equivalence of both methods.

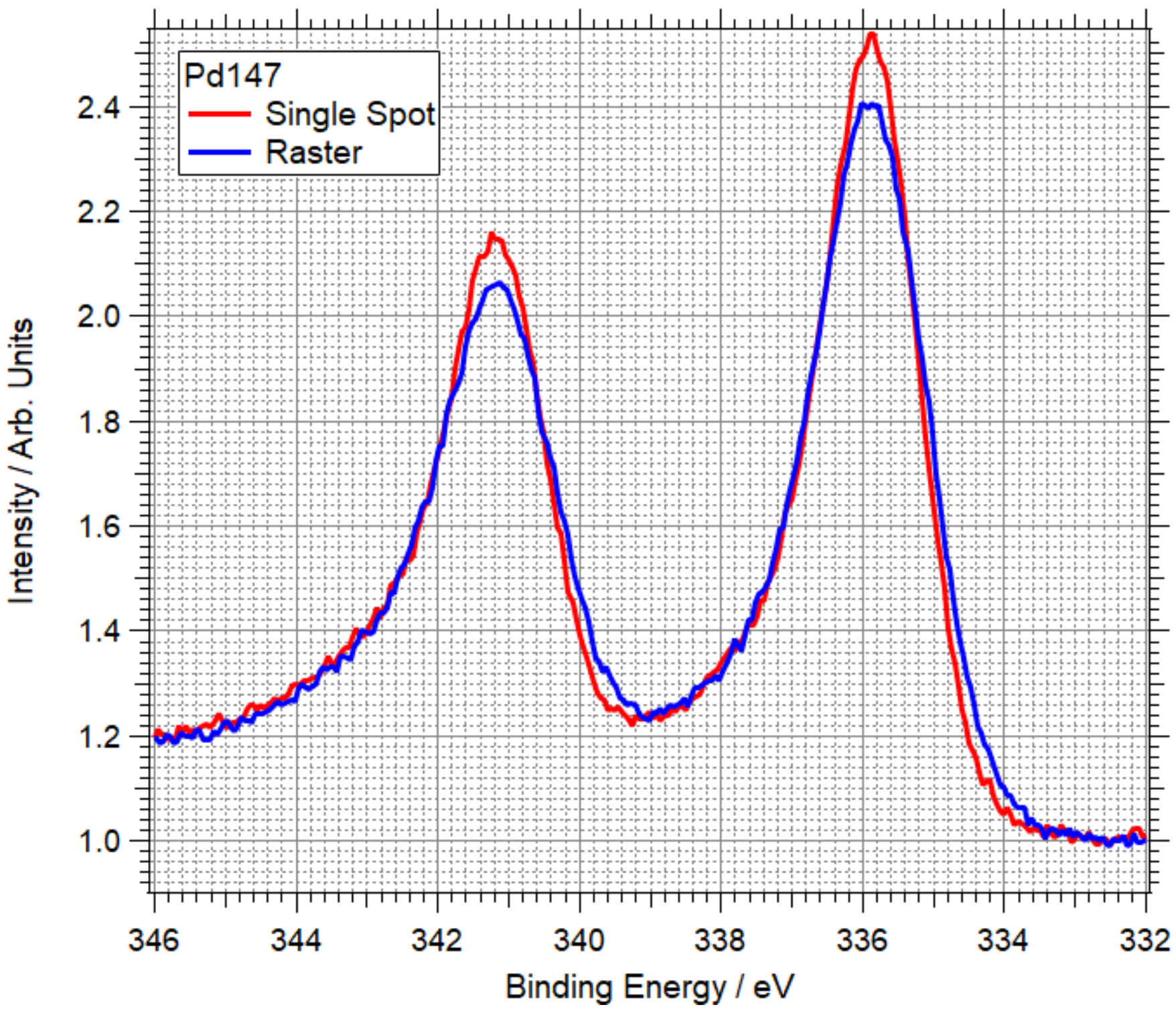

Figure SI 1: Pd 3d XPS spectra taken in vacuum prior to NAP-XPS experiments, for two Pd_{147}/Al_2O_3 samples. One sample had clusters deposited in a "single spot", whilst the beam was "rastered" over the other. The total Pd deposition was equal for both.

XPS Data Analysis

XPS Pd3d region data correction

Whilst the anodized alumina thin films are only a few nm thick, and broadly show minimal surface charging, there are still minor shifts seen in the XPS analysis. An example of this behaviour is shown in Figure SI 2 below, which comprises 40 scans of a Pd3d region taken consecutively. When uncorrected (left), it is clear that a progressive shift to lower binding energies is occurring. To ensure the spectra are clear enough to present, batches of 10 scans are combined to produce single spectra. As Al 2p scans were only typically taken either before or after the Pd3d region (typically after, with only a wide survey before), the best direct reference for charge correction is the latest Pd 3d scan available. As such, this is corrected based on an Al 2p peak position of 74.6eV. The other sets of Pd scans are then corrected to this peak position, and the iterations re-combined to produce a single, energy corrected Pd 3d wave. The right graphic shows a comparison of the original and “fixed” Pd3d spectra, with a shift of -0.27eV. Given the need for accuracy when dealing with small surface shifts as presented in this paper, these fixes are essential. This example shown is for the Pd_1 sample, though the same effect is seen for others. In most cases, only 4 scans were required, but the same principle is used, albeit on single scans rather than batches of 10.

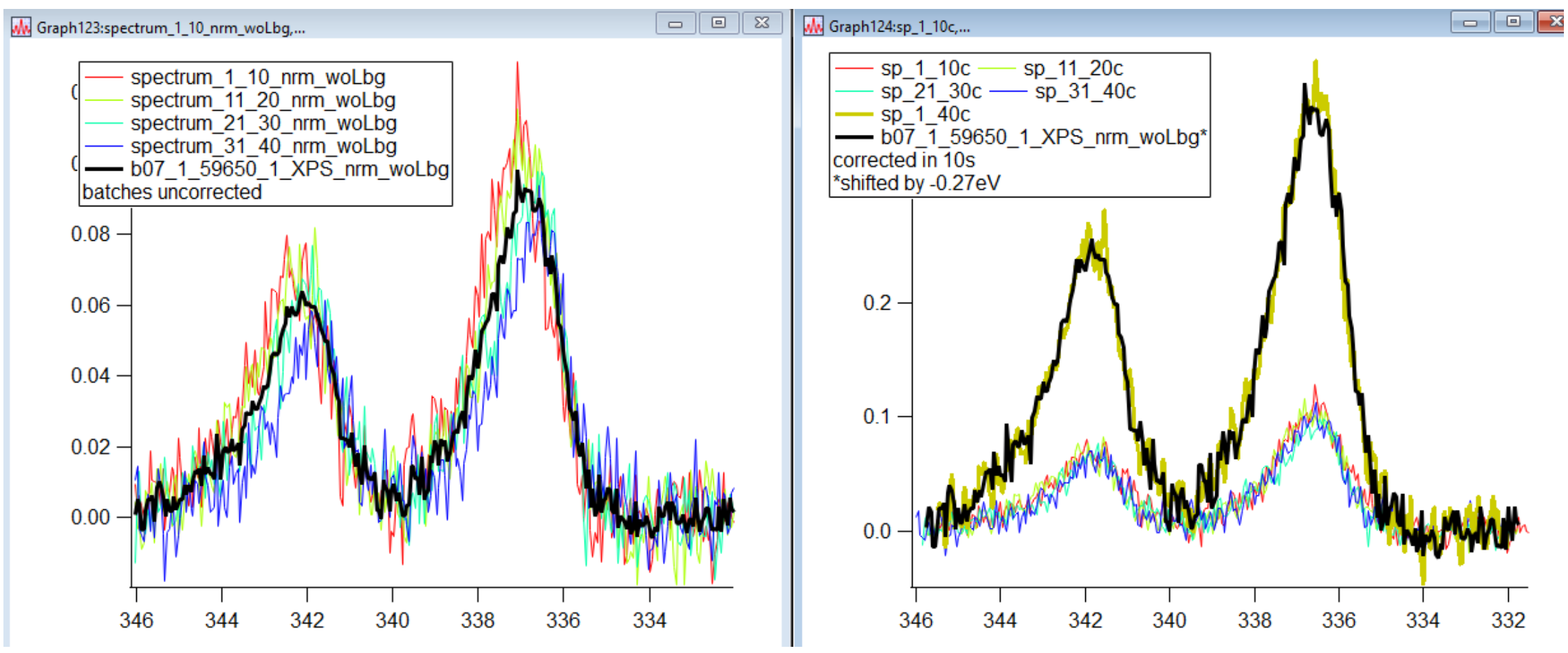

Figure SI 2: batches of individual Pd 3d XPS spectra taken of the Pd_1 sample. See text above for details.

Results

Al 2p vs Cluster size

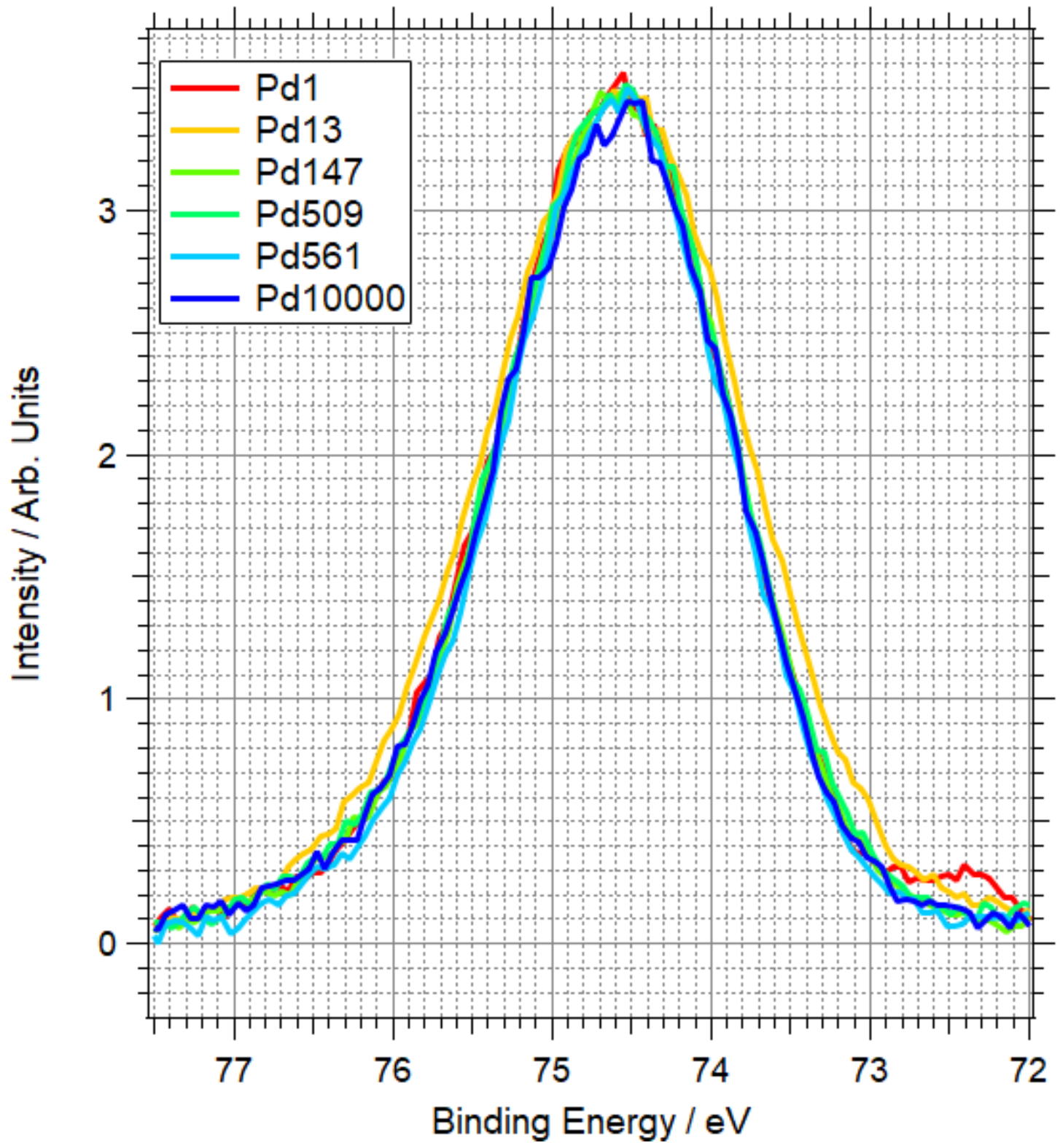

Figure SI 3: Al 2p region UHV XPS scans for samples of various sizes of Pd clusters on alumina film. Intensities rescaled in all cases to make shifts clearer. All spectra have been shifted to align at 74.6 eV.

Contamination

Figure SI 4 presents segments of XPS surveys for a range of cluster samples, to show levels of specific contaminants across samples. Samples with high levels of contamination were not considered in the data analysis presented in the main text.

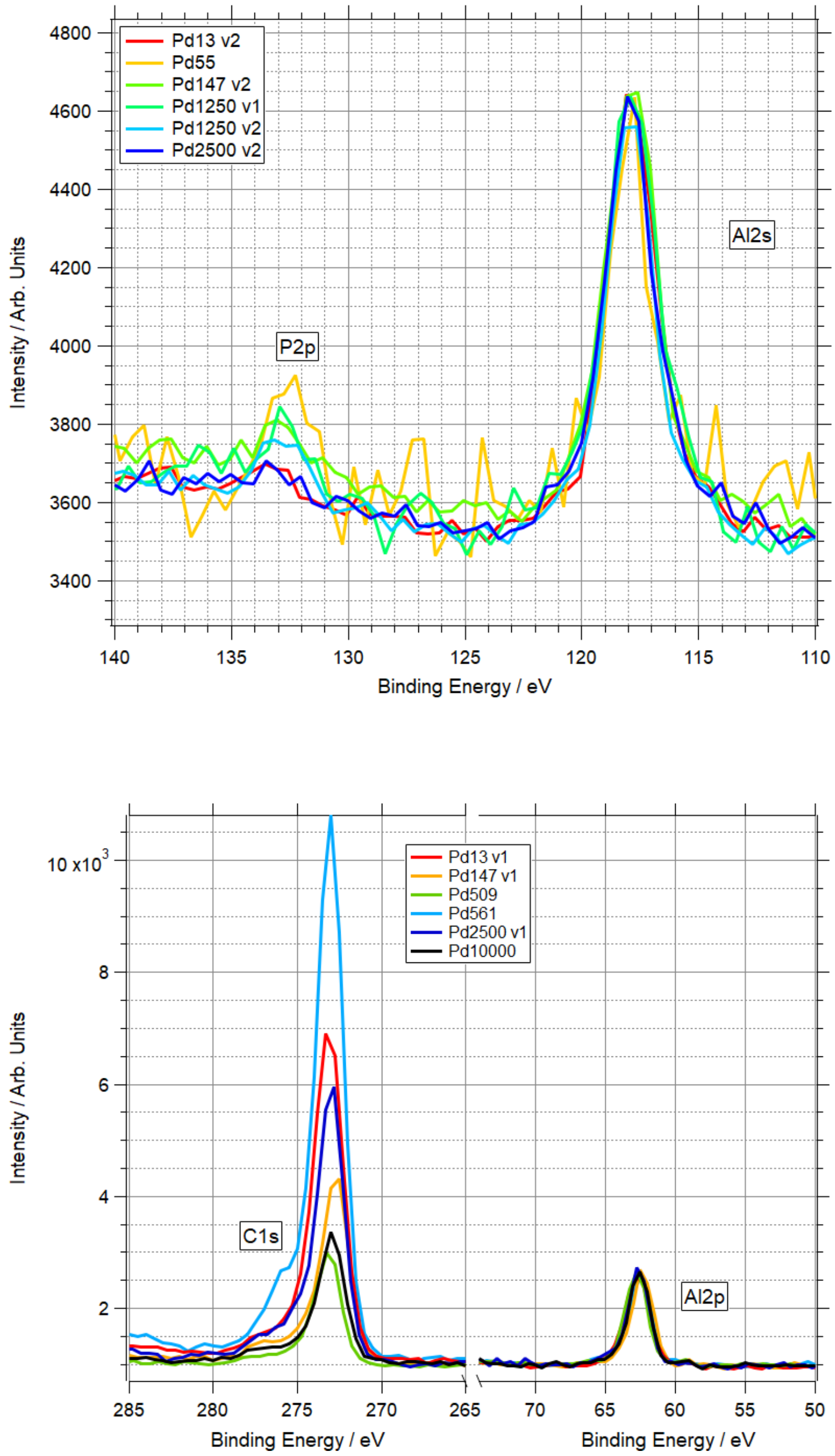

Figure SI 4: Segments of XPS surveys for a range of cluster samples, to show levels of specific contaminants across samples. Top: Al 2p and P 2p; bottom: C 1s and Al 2p
